# Fungal Pathogens Associated with *Tomicus* Species in European Forests: Regional Variations and Impacts on Forest Health

**DOI:** 10.3390/insects16030277

**Published:** 2025-03-06

**Authors:** Kateryna Davydenko, Denys Baturkin, Valentyna Dyshko, Jelena Lazarević, Adas Marčiulynas, Malin Elfstrand, Rimvydas Vasaitis, Audrius Menkis

**Affiliations:** 1 Department of Forest Mycology and Plant Pathology, Swedish University of Agricultural Sciences, 75007 Uppsala, Sweden; malin.elfstrand@slu.se (M.E.); rimvys.vasaitis@slu.se (R.V.); audrius.menkis@slu.se (A.M.); 2 Ukrainian Research Institute of Forestry and Forest Melioration, 61024 Kharkiv, Ukraine; valya_dishko@ukr.net; 3 Forest Protection Service “Kharkivlisozahyst”, 62458 Kharkiv, Ukraine; baturkin.denis@ukr.net; 4 Biotechnical Faculty, University of Montenegro, Mihaila Lalića 15, 81000 Podgorica, Montenegro; ena.lazarevic@gmail.com; 5 Lithuanian Research Centre for Agriculture and Forestry, Instituto al. 1, Akademija, LT-58344 Kėdainiai, Lithuania; adas.marciulynas@lammc.lt

**Keywords:** *Pinus sylvestris*, pine bark beetles, *Tomicus*, ophiostomatoid fungi, pathogens, fungal diversity, ITS2 rDNA, forest health, *Ophiostoma* spp., environmental factors

## Abstract

*Pinus species* are extensively abundant in Europe and, as pioneer trees, prominently influence local ecology. However, pine forests in Lithuania, Montenegro, and Ukraine have been significantly damaged by pine bark beetles (*Tomicus* sp.), which are closely associated with ophiostomatoid and other pathogenic fungi. This study aimed to identify the diversity of ophiostomatoid and other fungi associated with *Tomicus* sp. in these three countries. Fungi were isolated from beetles and identified. High-throughput sequencing of ITS2 rDNA yielded 285,828 reads, of which 91,141 high-quality reads were retained, representing 561 fungal operational taxonomic units (OTUs). The most important groups of fungi included ophiostomatoids, yeasts, and plant pathogens. While the fungal communities associated with *Tomicus* spp. were influenced more by environmental factors than by beetle species, the presence of known pathogens such as *Ophiostoma* spp. indicates that *Tomicus* spp. could play a significant role in dispersing harmful fungi. Although the virulence of these fungi may vary, their association with potentially pathogenic species suggests that *Tomicus* spp. may contribute to forest health decline, especially if environmental conditions or host susceptibility change.

## Simple Summary

This study explored the relationship between pine bark beetles, *Tomicus* spp. and fungi in forests of three European countries (Lithuania, Ukraine, Montenegro) with different climates. Bark beetles are tiny insects that attack trees, often carrying fungi that can harm forests. The study aimed to understand the types of fungi associated with these beetles and their potential impact on forest health. The results revealed significant differences in fungal communities across the regions, influenced by local environmental conditions and tree species. Some fungi, such as those causing blue-stain or shoot blight, are harmful to tree health and were found in high numbers in certain areas. Interestingly, some fungi that typically support beetle development were also discovered, showing how these fungi–beetle interactions can be both beneficial and harmful. The findings highlight the role of these beetles in spreading harmful fungi, especially as climate change weakens trees and creates favorable conditions for beetles. This research is valuable for understanding how beetles and fungi interact and showing the risks they pose to forests. It provides important information for forest management strategies to protect trees from these threats, especially in the face of environmental changes that could exacerbate the problem.

## 1. Introduction

From an economic and ecological perspective, Scots pine (*Pinus sylvestris* L.) stands as one of the most significant tree species in Europe, known for its broad ecological adaptability across its native range. *Pinus halepensis* Mill. is a crucial forest tree in the Mediterranean region, providing biodiversity hotspots, soil and water protection, carbon sequestration, and landscape quality [1]. It also aids in soil preparation for other native species. However, both *P. sylvestris* and *P. halepensis* face threats from prolonged droughts, forest fires, diseases and pests, particularly bark beetles. Climate change exacerbates these challenges, with rising temperatures enhancing bark beetle survival and development, potentially impacting the long-term health and vitality of these forests.

The interaction between bark beetles and microorganisms is a major driver of tree damage globally [2,3,4]. Bark beetles, often associated with various microorganisms, transmit fungi that provide nutrients and weaken tree defenses, benefiting both the beetles and the fungi [5,6]. These beetles are particularly linked to ophiostomatoid fungi, which can cause significant damage to *Pinus* and other tree species worldwide [2,7].

Bark beetles of the genus *Tomicus* (Scolytinae, Coleoptera, Curculionidae) are significant forest pests capable of causing extensive damage to pine trees, particularly during outbreaks [4,8]. Adult beetles bore galleries beneath the bark for breeding, while their larvae excavate lateral tunnels, collectively posing a serious threat to forest health. Although many bark beetle species primarily infest *Pinus* spp. and are often considered secondary colonizers targeting dead, diseased, or weakened trees, certain *Tomicus* species stand out for their capacity to kill healthy trees during periodic outbreaks. Of the six known species, *T. piniperda*, *T. destruens*, and *T. minor* occur in Europe, with *T. piniperda* now widely distributed across Eurasia and introduced to North America [9,10,11].

Among more than 10 bark beetle species that attack *P. halepensis*, only *Pityogenes calcaratus*, *T. destruens*, and *Orthotomicus erosus* are recognized as significant pests [11,12,13].

The ecology of *Tomicus* pine bark beetles is complex, involving interactions with host trees, environmental conditions, and symbiotic relationships with fungi. Bark beetles associated with pine have long carried blue-stain fungi, which were not considered pathogenic until an outbreak in central France that caused significant pine mortality. This outbreak implicated *T. piniperda* and *Ips sexdentatus* (Börner), along with their fungi [14]. The ability of beetles to carry and introduce blue-stain fungi exacerbates the damage to pine trees by impeding water and nutrient transport, resulting in reduced timber value [6,15,16]. Moreover, these beetles can contribute to the spread of other aggressive pine pathogens. For instance, *T. piniperda* has recently been identified as a plausible vector of *Fusarium circinatum* in Spain, *Sydowia polyspora* in Italy and Portugal, and *Diplodia sapinea*, which has been found on various insects but is primarily associated with *T. piniperda* in several European regions, including Spain and Italy [17,18,19,20].

The invasion patterns of fungi in the sapwood of trees attacked by bark beetles vary depending on the insect species, host tree species, and environmental factors. In Europe, *T. piniperda* is a well-known vector for numerous ophiostomatoid fungi, with *Ophiostoma minus* and *Leptographium wingfieldii* being dominant species in many countries [8]. While the mycobiota associated with *T. piniperda* has been extensively studied in Sweden, Poland, and Spain [8,17,21], significant gaps remain in understanding fungal invasion patterns in trees attacked by *T. piniperda* in other European regions.

In contrast, the association between *T. destruens* and fungi of the genus *Leptographium* has been thoroughly investigated in *Pinus pinea* and *P. pinaster* forests in Tuscany, central Italy [22]. Additionally, *T. piniperda* and *T. destruens* have recently been investigated in *Pinus radiata* plantations infected by *F. circinatum* [18]. However, there remains a notable lack of data on fungi transmitted by bark beetles in the Mediterranean region, particularly in association with *P. halepensis*.

These findings emphasize the need for further research on fungal associations with bark beetles in diverse ecosystems, particularly regarding their roles in pathogen transmission and their impacts on forest health. This study aimed to fill this gap by documenting and comparing the species composition and frequency of fungi associated with pine bark beetles attacking principal pine trees in central or continental Europe (represented by Ukraine and Lithuania) and the Mediterranean/southern region (represented by Montenegro). We hypothesize that fungal communities associated with *Tomicus* species are strongly influenced by local environmental conditions and host tree species, resulting in significant regional differences in fungal composition and potential pathogenic impacts.

## 2. Materials and Methods

### 2.1. Site Characteristics and Sample Collection

**Figure 1:**
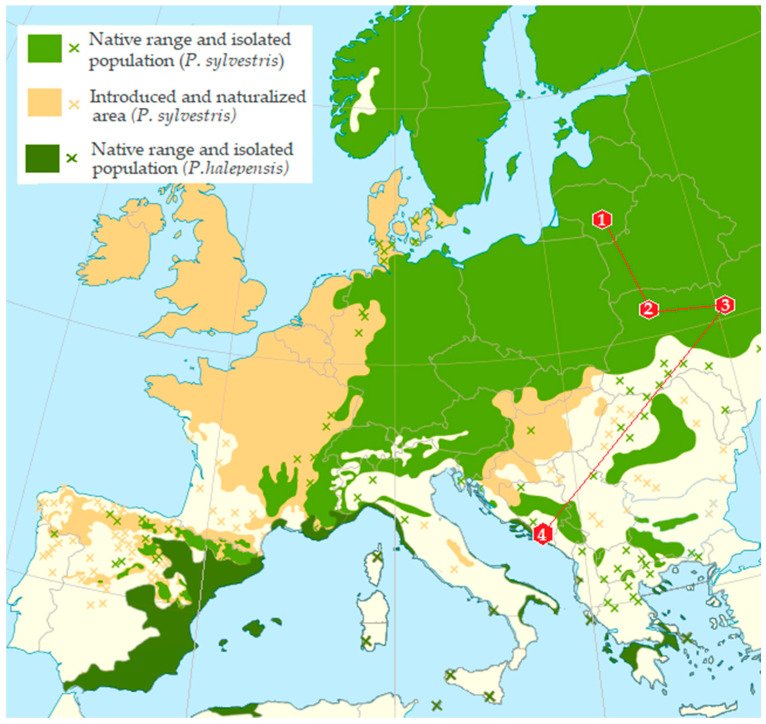
Map of Europe showing the distribution of *Pinus sylvestris* and *Pinus halepensis* and the 4 sampling sites. Coding for the sampling sites is given in Table 1.

Adults of bark beetles from infested branch and trunk sections were used to analyze the mycobiota. Beetle samples were collected over a one-day period during the winter between 15th January and 1st March 2018, in each of the sampling plots from *P. sylvestris* in Lithuania (LT) and Ukraine (UA) and from *P. halepensis* in Montenegro (MN) (Table 1, Figure 1). The distance between sampled trees within each plot ranged from 50 to 150 m. The sampling sites covered an area of approximately up to 3 ha, depending on the country and location. The distances between sampling localities were as follows: 347 km between two sites in Ukraine (UA), 1317 km between Lithuania (LT) and Ukraine (UA), 989 km between Ukraine (UA) and Montenegro (MN), and 1897 km between Lithuania (LT) and Montenegro (MN). A total of 180 beetles were collected. All sampling plots exhibited favorable conditions for *Tomicus* spp.

Samples from Ukraine and Lithuania were collected from pine shoots and felled pine trees, which were laying on the forest floor and colonized by pine bark beetles. In Montenegro, samples were collected from stumps or damaged pine trees after removing bark particles. Insects from Ukraine and Lithuania were identified as *T. piniperda*, while insects from Montenegro were identified as *T. destruens*. Historically, *T. destruens* has been confused with *T. piniperda* due to their morphological similarities, with early studies suggesting that *T. destruens* may be a Mediterranean ecotype of *T. piniperda*.

Insects inside shoots and under bark were removed using sterilized tweezers and stored at 4 °C. Each bark beetle was transferred to a single sterilized tube and immersed in 100 µL of Tween 80 (PanReac Química, Barcelona, Spain) at 1% *v*/*v*.

Sampling sites:

- *Lithuania*

Located in Central Europe, Lithuania has a temperate climate with distinct seasons. Forests cover 33.5% of the country, with varying tree species dominance: *P. sylvestris* in the southeast, *Picea abies* in the west, and deciduous trees in the north-central regions. Lithuania experiences a temperate continental climate with cold winters and mild summers, characterized by moderate precipitation throughout the year, particularly during summer months.

- *Montenegro*

The forest ecosystems of the Montenegrin Adriatic coast cover 49.9% of the region, mainly consisting of low forests, degraded stages, and shrubs. These forests play a crucial ecological role in soil erosion control, water protection, and biodiversity preservation. Coastal forests, especially those with *P. halepensis*, are highly vulnerable to frequent fires, which spread quickly and severely damage vegetation and soil, making recovery difficult. Montenegro has a Mediterranean climate along the coast, with hot, dry summers and mild, wet winters, while inland areas experience a more continental climate with colder winters and hotter summers.

- *Ukraine*

Sampling was conducted in the flat part of the country’s territory in the forest zone (Polissja), where forests cover 37% of the area. Pine forests account for 42% of the total forest area, with 32% dominated by *P. sylvestris*, distributed across much of the country’s flat land. Ukraine experiences a continental climate with cold winters and warm summers, featuring significant regional variation—from a humid subtropical climate in the south to a more continental climate in the north. Precipitation is mostly concentrated in spring and summer.

### 2.2. DNA Extraction, Amplification, and Sequencing

For the identification of fungal communities, beetles from Ukraine, Montenegro, and Lithuania (Table 1), sampled during field trapping and/or from wood, were used for DNA analysis. Genomic DNA was isolated separately from 48 adults from Lithuania, 96 from Ukraine, and 36 from Montenegro. Before DNA extraction, insects were individually placed in 1.5 mL centrifugation tubes and lyophilized at −60 °C for one day without surface sterilization. Lyophilized insects were then placed into 2 mL screw-cap tubes along with two glass beads and homogenized using a FastPrep instrument (Precellys 24; BertinTechnologies, Rockville, MD, USA). DNA extraction was performed using the 3% CTAB protocol as described in Menkis [23].

The concentration of genomic DNA was determined using a NanoDrop™ One spectrophotometer (Thermo Scientific, Rochester, NY, USA) and adjusted to 10 ng/µL. Amplification of the ITS2 rDNA region was completed using barcoded primers gITS7 and ITS4, following the protocol by Clemmensen et al. (2016) [24]. PCR amplification was carried out in 50 μL reactions using an Applied Biosystems 2720 thermal cycler (Foster City, CA, USA). The PCR protocol consisted of an initial denaturation at 94 °C for 5 min, followed by 30 cycles of 94 °C for 30 s, annealing at 56 °C for 30 s, and extension at 72 °C for 30 s, ending with a final extension at 72 °C for 7 min.

PCR products were assessed on 1% agarose gels stained with GelRed (Biotium, Fremont, CA, USA). The PCR products were purified using a mixture of 3 M sodium acetate (pH 5.2) (Applichem GmbH, Darmstadt, Germany) and 96% ethanol (1:25 ratio). Cleaned PCR products were quantified using a Qubit Fluorometer 4.0 (Life Technologies, Stockholm, Sweden) and then pooled in an equimolar mix. Sequencing was performed on the PacBio RSII platform using two SMRT cells at the SciLifeLab in Uppsala, Sweden.

### 2.3. Bioinformatics

The SCATA NGS sequencing pipeline (http://scata.mykopat.slu.se, accessed on 20 May 2024) was used for sequence quality control and clustering. The procedures included the removal of primer dimers, short sequences (<200 bp), low-quality sequences (Q < 20), and homopolymers, which were collapsed to 3 base pairs (bp) before clustering. Sequences lacking a tag or primer were removed, but sample information was retained as metadata. Single-linkage clustering based on 98.5% similarity was used to cluster different operational taxonomic units (OUTs).

OTUs were assigned taxonomic names using the GenBank (NCBI) database and the Blastn algorithm. The criteria for assignment included sequence coverage >80%, similarity to the genus level at 94–97%, and similarity to the species level at 98–100%. Representative sequences of fungal nonsingletons, as part of the Targeted Locus Study project, have been deposited in GenBank under the accession number (KIEZ00000000). Taxonomical information was also associated with each cluster using the SH mapping feature of the UNITE database (https://unite.ut.ee/analysis.php, accessed on 20 April 2024).

### 2.4. Statistics

A non-parametric chi-square test was used to assess differences in the relative abundance of common fungal OTUs associated with different countries. For multiple comparisons between two locations within a country, confidence limits for *p*-values from the chi-square tests were adjusted using the Bonferroni correction.

The detrended correspondence analysis (DCA) in Canoco 5 was used to characterize the composition of the fungal communities [25].

A Venn diagram was constructed to visualize the overlap and unique distribution of operational taxonomic units (OTUs) among the three study sites (LT, MN, UA). OTUs were identified using the presence/absence data of fungal species in each sample. The analysis was performed using the VennDiagram package in R 4.1.1 (https://www.r-project.org, accessed on 24 December 2024).

Indicator species analysis was performed to identify fungi strongly associated with specific environmental conditions. The analysis was conducted using the indicspecies package in R 4.1.1 (https://www.r-project.org, accessed on 16 December 2024), which calculates indicator values based on species’ relative abundance and distribution patterns within predefined groups (e.g., different environmental conditions or locations). The analysis was performed using the multipatt function from the indicspecies R package with 999 permutations. Non-metric multidimensional scaling (NMDS) was used to analyze the differences in fungal community composition across three sites (LT, MN, and UA). The analysis was performed using the metaMDS function from the vegan package in R, with the Bray–Curtis dissimilarity index as the distance measure to quantify the dissimilarity between samples based on their fungal community composition. To test the significance of the observed community differences across the three sites, a permutation-based analysis of variance (PERMANOVA) was conducted using the adonis2 function from the vegan package, with the site as the grouping factor. The PERMANOVA test indicated a significant difference in fungal community composition between the sites (*p* < 0.001). All analyses were performed using Vegan 2.5.7 and Stats 3.6.2 in R 4.1.1 (https://www.r-project.org, accessed on 24 December 2024) [26].

## 3. Results

High-throughput sequencing of fungal communities generated 285,828 reads, of which 91,141 high-quality reads were retained, while low-quality reads, non-fungal OTUs, and singletons were excluded. This sequencing revealed diverse fungal taxa, with 561 operational taxonomic units (OTUs) identified.

**Figure 2:**
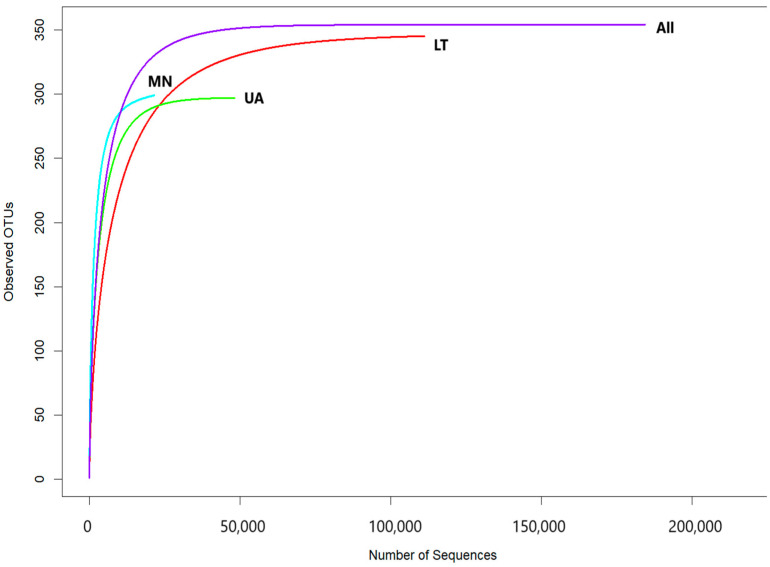
Rarefaction curves showing the relationship between the cumulative number of fungal OTUs and ITS2 rDNA sequences from each country (LT—Lithuania; MN—Montenegro; UA—Ukraine) as well as all data combined.

The rarefaction curves, showing the relationship between the number of reads and operational taxonomic units (OTUs) in each sample, reached a stable plateau as the sample size increased (Figure 2). These results indicated that the sequencing depth and the number of OTUs were sufficient for each sample to accurately represent the fungal communities, allowing for further analyses (Figure 2).

The most common fungi obtained from beetles were primarily plant pathogens (46.7%) or saprotrophic fungi (43.3%) including primary, secondary and tertiary decomposers, belonging to either *Ascomycota* (80%) or *Basidiomycota* (20%). The saprotrophic group includes also lignocellulose-degrading saprotrophs, which are common in soil and fallen plant litter. Several plant pathogens with the potential to cause disease outbreaks were identified. In this study, *Cladosporium* spp., *Ophiostoma* spp., and *D. sapinea* were the most prevalent fungi, accounting for 21.5%, 20.6%, and 17.2% of the fungal community, respectively. *Cladosporium* sp. was found in every sample group, showing a notably higher presence on the surface of *T. piniperda* beetles, regardless of their origin (Table 2).

**Figure 3:**
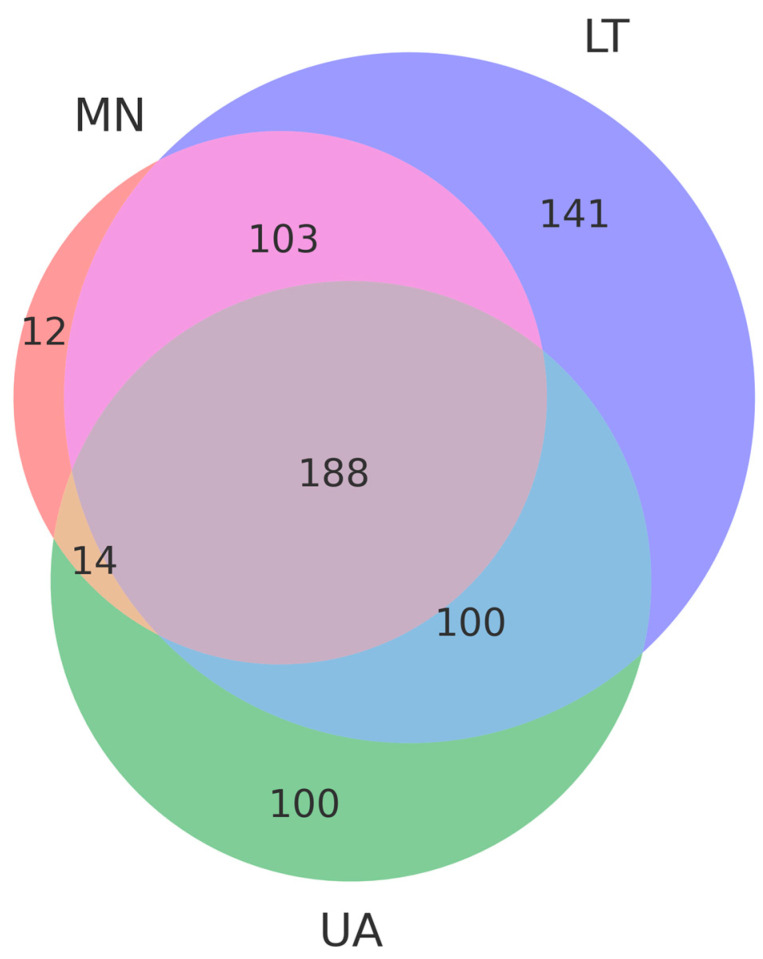
The Venn diagram, based on operational taxonomic units (OTUs), represents the common and unique OTUs in each group, showing the distributional differences in fungi among the three countries: Lithuania (LT), Montenegro (MN) and Ukraine (UA). Violet, salmon and green colors indicate unique OTUs in Lithuania (LT), Montenegro (MN) and Ukraine (UA) respectively. Rose color indicates common OTUs in Lithuania (LT) and Montenegro (MN), blue color indicates common OTUs in Lithuania (LT) and Ukraine (UA), beige color indicates common OTUs in Montenegro (MN) and Ukraine (UA). Dusty purple color Montenegro (MN), blue color indicates common OTUs among the three countries: Lithuania (LT), Montenegro (MN) and Ukraine (UA).

A total of 561 operational taxonomic units (OTUs) were selected from three pooled groups—Lithuania (LT), Montenegro (MN), and Ukraine (UA)—to compare species with a relative abundance of more than 3%. Moreover, 14 OTUs were common to MN and UA samples, 100 to UA and LT samples, and 103 to LT and MN samples, respectively (Figure 2). As depicted in the Venn diagram, 188 common OTUs were present in all samples, suggesting significant differences in fungal communities among the three areas due to varying ecological conditions (Figure 3).

While various *Ophiostoma* species were the most common in all countries, the relative abundance of other pathogens varied significantly. The important needle and shoot pathogen, *D. sapinea*, was among the most common fungi (17.2%), found on the beetle surface in all three groups. It was the most common species in Ukraine (34.2% of all samples) and one of the most common in Montenegro (14.5% of all samples). In Lithuania, only 2.8% of all *T. piniperda* beetles were associated with *D. sapinea*.

In Montenegro, the most common pathogen was *Fusarium oxysporum* (18.8%), whereas this fungus was rare on beetles in Lithuania (0.3%) and Ukraine (0.01%). *Alternaria alternata* was relatively common in Lithuania (11.3%) and less common in Ukraine (0.8%), but it was not found on beetles from Montenegro. These data confirm significant differences in the fungal communities associated with pine bark beetles across different countries.

Our results showed that ophiostomatoid fungi were distributed among three genera in two orders: *Ophiostoma* sensu lato and *Leptographium* s. l. in the *Ophiostomatales*, and *Graphium* and *Ceratocystis* in the *Microascales* (Table 3). The most commonly encountered taxa belonged to *Ophiostoma* s. l. All *Ophiostoma*, *Ceratocystis*, *Graphium*, and *Leptographium* species were identified and assigned at the genus level, as species-level identification requires the use of specific primers (e.g., β-tubulin gene and the partial elongation factor 1-alpha (EF1-α) gene).

Interestingly, *Geosmithia* sp. was found to be associated with pine shoot beetles only in Ukraine. *Geosmithia* is considered a ubiquitous fungal symbiont associated with wood-boring bark beetles. Most *Geosmithia* species are saprophytic and do not harm host trees, except for the canker-causing *Geosmithia morbida*, which affects walnut trees.

High species richness of fungal pathogens associated with pine shoot beetles was observed at each site, primarily belonging to either *Ascomycota* or *Basidiomycota*. Several fungal pathogens with the potential to infect pine stands were detected in each country (Table 2). We emphasize the potential of important fungal pathogens to cause diseases in nursery-grown tree seedlings. Although *Botrytis cinerea* is a well-known nursery pathogen causing outbreaks in forest nurseries, it was found in all groups.

**Figure 4:**
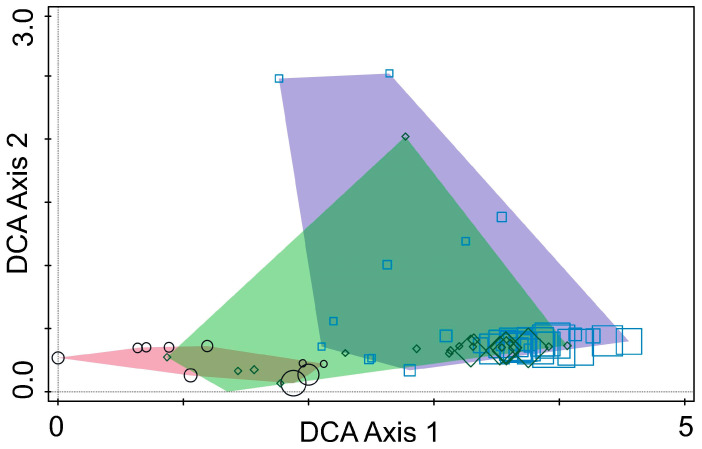
Ordination diagram based on detrended correspondence analysis of fungal communities from *Tomicus* species collected in Lithuania (polygon—purple; points—squares), Montenegro (polygon—rose; points—circles), and Ukraine (polygon—green; points—diamonds). The size of individual points (squares, diamonds or circles) represents the relative richness of fungal OTUs.

DCA identified clear separation among fungal communities from the three regions, indicating significant regional variation in fungal composition (Figure 4). DCA Axis 1 captured the primary differences in fungal community composition across the three countries, while Axis 2 reflected ecological differences. The clustering patterns and regional separations showed that fungal communities are strongly influenced by geographic location, environmental conditions, and host tree species. This shows the importance of local ecological factors in shaping fungal communities associated with pine bark beetles.

**Figure 5:**
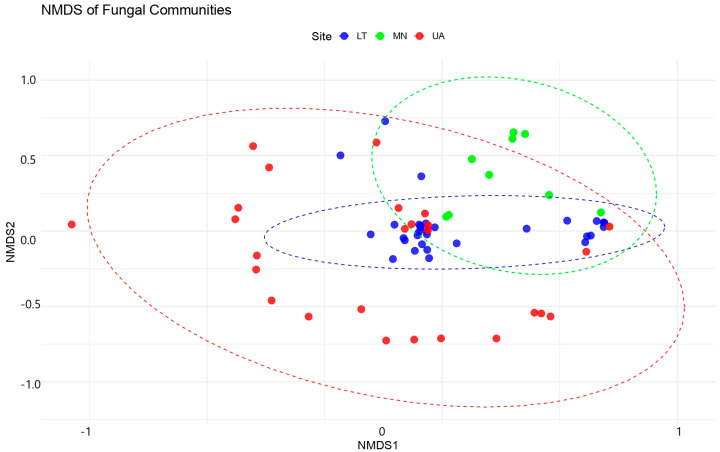
Non-metric multidimensional scaling (NMDS) plot of the fungal community. NMDS of dissimilarity based on a Bray–Curtis distance matrix of rarefied fungal OTU abundances. The dashed lines on the NMDS plot indicate the relative positioning of fungal communities in ordination space. The values (−1, 0, 1 for NMDS1 and −1, −0.5, 0, 0.5, 1 for NMDS2) correspond to the ordination scores, which reflect differences in fungal community composition based on Bray–Curtis dissimilarity. Closer points represent more similar communities, while greater distances indicate higher dissimilarity.

The NMDS analysis produced a 2D ordination with a stress value of 0.1045, indicating a good representation of the original community dissimilarities in the reduced space (Figure 5).

PERMANOVA analyses revealed significant differences in fungal community compositions between sites, although model explains only about 28.39% of the variation in fungal community composition across the three sites (LT, MN, UA). While this is a small proportion, the differences between the sites are statistically significant (*p* = 0.001).

The F-statistic (2.9769) suggests that the sites are different from each other in terms of fungal community composition. Ultimately, 71.61% of the variation remains unexplained within the sites, meaning the communities within each site have substantial variation. Since the *p*-value is highly significant (*p* = 0.001), we can conclude that there are indeed significant differences in fungal communities between the three sites.

**Figure 6:**
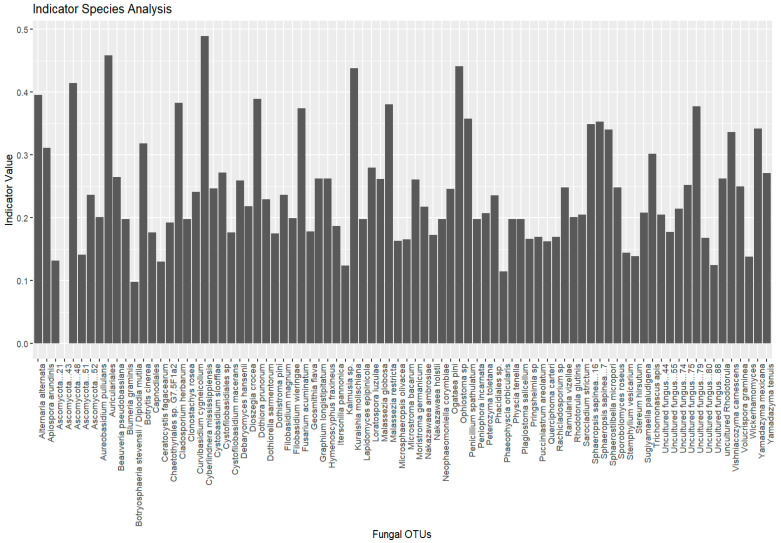
Indicator species analysis results for fungal communities of *Tomicus* species collected in Lithuania, Montenegro, and Ukraine.

Indicator species analysis identified fungal species significantly associated with specific environmental conditions. A total of 755 species were initially considered, with 27 species selected as indicator species, based on their significant association with distinct groups (Lithuania, Montenegro, and Ukraine). No indicative species were found to be associated with multiple groups (Figure 6).

The plot shows the top 20 most significant indicator species (*p* < 0.05) identified for different groups (e.g., location, host tree, beetle species). Each bar represents a fungal OTU. The height of the bars corresponds to the indicator value, which reflects the strength of association between the species and the given group. Species with higher indicator values and lower *p*-values are considered strong indicators of specific groups.

Group Lithuania (19 species): The analysis revealed several fungal species with strong associations to Lithuania, with indicator values (statistic values, *p*-values) indicating significant associations (*p* ≤ 0.05) with specific species. Notable species included *Auriculariales* (stat = 0.458, *p* = 0.002), *Ophiostoma* sp. (stat = 0.442, *p* = 0.002), and *Ascomycota* (stat = 0.414, *p* = 0.001), all showing strong associations with Lithuania. Other species, such as *Alternaria alternata* and *Dothiora prunorum*, also showed significant associations with *p*-values ≤ 0.005. A number of species were found to be significant indicators for this group, with effect sizes decreasing as their associations became less pronounced.

Group Montenegro (five species): In Montenegro, five species were identified, with *Cyberlindnera mississippiensis* (stat = 0.489, *p* = 0.001) and *Kuraishia molischiana* (stat = 0.438, *p* = 0.001) demonstrating the highest association values. *Fusarium acuminatum* (stat = 0.375, *p* = 0.006), and *Sphaerostilbella micropori* (stat = 0.340, *p* = 0.010) also showed significant associations, suggesting that these species play a key role in the microbial community of this region.

Group Ukraine (three species): In Ukraine, only three species were significantly associated with the Ukrainian group: *Botrytis cinerea* (stat = 0.319, *p* = 0.012), *Isaria* sp. (stat = 0.282, *p* = 0.033), and *Sporobolomyces roseus* (stat = 0.319, *p* = 0.012). These species represent unique ecological markers for the conditions in Ukraine.

## 4. Discussion

This study provided valuable insights into the fungal communities associated with *Tomicus* spp. in three European countries with different climates, showing significant regional differences in fungal composition and potential pathogenic impacts. These findings emphasize the importance of ecological context and environmental variation in shaping fungal–beetle interactions. Differences in fungal communities between countries may also be influenced by the host species, with *P. sylvestris* in Ukraine and Lithuania and *P. halepensis* in Montenegro likely contributing to the observed variability. High-throughput sequencing revealed a diverse fungal community, with 561 OTUs identified and 188 shared across all regions, indicating ecological variability influenced by local conditions. Rarefaction analyses confirmed adequate sequencing depth, ensuring comprehensive representation of fungal diversity in all samples.

Ophiostomatoid fungi, known for their associations with bark beetles, were prominent, with *Ophiostoma* sp. being the most common blue-stain species, while *Leptographium* spp., *Graphium* sp., and *Ceratocystis* sp. were less frequent. Our findings align with studies from Poland, Spain, Sweden, and Norway [21,27,28,29], which have reported numerous ophiostomatoid fungi linked to bark beetles. However, contrasting earlier reports of *T. piniperda* as a vector for highly pathogenic fungi such as *O. minus* and *Leptographium* spp., we could not confirm the presence of *O. minus* due to the need for specific identification techniques [14,22,30].

Interestingly, *Ambrosiella hartigii*, a mycangial symbiont of ambrosia beetles like *Anisandrus dispar*, was detected in Lithuania in association with *T. piniperda*. Symbiotic fungi such as *Ambrosiella* and *Raffaelea* play a critical role in beetle development, providing essential organic compounds [31,32]. While generally harmless to trees, these fungi can disrupt water and nutrient flow in sapwood, indirectly affecting tree health. Some species, such as *Raffaelea lauricola*, are highly pathogenic and cause severe tree mortality [32]. The detection of *A. hartigii* with *T. piniperda* underscores the complexity of fungal–beetle interactions and suggests potential new ecological roles for this fungus outside its typical hosts. Among the ascomycetes detected, potentially pathogenic species such as *A. alternata*, *B. cinerea*, *Dothistroma* sp., *F. oxysporum*, *Stemphylium vesicarium*, and *D. sapinea* were prominent. *Dothistroma* sp., a needle pathogen, was found only in Montenegro [33], while *B. cinerea*, a known nursery pathogen, was common across all regions.

*Diplodia sapinea*, responsible for shoot blight and dieback in *P. sylvestris*, was prevalent across all sample groups. Its high prevalence, particularly in Ukraine and Montenegro, raises concerns about its potential role during both maturation feeding and larval development. Reports of *D. sapinea*-related disease have increased over the past decades, often in association with stressed trees and bark beetles such as *T. piniperda*, *Hylastes attenuates*, and *Hylurgops palliates* [34]. In Lithuania and Ukraine, *Beauveria pseudobassiana*, a dual-role saprotroph and endophyte, was one of the most abundant isolates. Its ability to activate plant defenses aligns with its potential in biocontrol applications. Additionally, *Cladosporium* spp., commonly found on living and dead plant material, were frequently detected, consistent with their status as prevalent environmental molds.

Our findings align with research that has noted weak fidelity between phloem-feeding bark beetles and their associated fungi. However, fungal communities within beetle mycangia are typically more stable [7]. While previous studies suggested that fungal communities are shaped more by beetle species than geographic location [7,35], our results indicate the opposite. For *T. piniperda*, which lacks mycangia, fungal communities are more strongly influenced by geographic location, host tree species, and environmental conditions than by beetle species. This observation is consistent with a study conducted in southwestern China, which showed that the epibiotic fungal community structures of three *Tomicus* species were strongly conditioned by the locations and pine hosts, but weakly by beetle species and infection sections [36]. These findings support the view that, in species without mycangia, environmental factors have a stronger influence on fungal communities than the beetle species themselves.

The presence of different *Tomicus* species (e.g., *T. piniperda* in Ukraine and Lithuania, and *T. destruens* in Montenegro) further contributes to regional differences. For a long time, *T. piniperda* and *T. destruens* were considered synonymous; however, they were later differentiated based on morphological features. Despite this distinction, earlier studies noted intermediate forms and variations, complicating their identification [12,13]. Given their close relationship, both species likely share similar ecological traits, including life cycle and host utilization. These ecological similarities may contribute to overlapping fungal associations, while regional factors and host tree differences drive the observed variation in fungal communities.

The indicator species analysis revealed distinct fungal communities associated with each group, highlighting the regional variability in fungal composition. The Lithuanian group demonstrated a rich diversity of fungal species with strong associations, particularly among species such as *Auriculariales*, *Ophiostoma* sp., and *Ascomycota*. These species may be indicative of the environmental conditions and ecological processes prevalent in the Lithuanian ecosystem. The Montenegrin and Ukrainian groups both showed a more limited set of fungal species but still revealed significant associations, notably *Cyberlindnera mississippiensis* and *Kuraishia molischiana*, suggesting these fungi may have an important role in the microbial landscape of this Mediterranean region. In contrast, Ukraine’s group was represented by only three species, *Botrytis cinerea, Isaria* sp., and *Sporobolomyces roseus* which were found to be a significant indicator of the specific environmental and host tree conditions in this region.

While ITS2 sequencing provided a broad overview of fungal diversity, this marker has inherent limitations in resolving closely related fungal species, particularly within certain taxonomic groups. Future studies should consider additional markers (e.g., TEF1-α or β-tubulin) for improved species resolution.

These findings provide valuable insights into the fungal community structure in different geographical regions and highlight the ecological roles of indicator species in relation to their environmental conditions. The results suggest that local environmental factors play a critical role in shaping fungal communities, and that certain fungi are highly specific to particular regions.

It is important to note that the sampling design introduced unavoidable variations, such as differences in host tree species, beetle species, and collection methods, which may have influenced the observed fungal communities. While these variations limit the ability to draw direct comparisons, they also reflect real-world ecological interactions, where fungi interact with multiple hosts and beetle species across heterogeneous environments. The influence of different beetle life stages, particularly supplementary feeding in shoots versus breeding under bark, should be further explored in future studies to understand their role in shaping fungal communities.

This study confirms that bark beetles are significant vectors of forest pathogenic fungi, raising concerns about the introduction and spread of alien diseases. While some bark beetles can kill healthy trees, fungi often assist by overcoming plant defenses, causing economic losses in forest plantations. Although this study focused on fungal diversity and some of the identified fungi have been previously described as plant pathogens, our study does not confirm their virulence or direct impact on tree health. The presence of potentially pathogenic fungi in association with bark beetles suggests possible ecological roles, but additional cultivation-based studies and pathogenicity assays are required to determine their actual effects on forest ecosystems. Future research should incorporate isolation techniques and controlled infection experiments to assess the pathogenic potential of key fungal species.

Climate change, with predicted warming in Europe, may exacerbate these issues by increasing bark beetle generations and weakening host trees. However, the pine shoot beetle may remain limited to one generation per year due to photoperiod and low-temperature requirements. Understanding the complex interactions between beetles, fungi, and environmental factors is critical for effective forest management. Continued monitoring and research are necessary to develop strategies for mitigating the risks posed by these interactions, particularly in the context of climate change and shifting host dynamics.

## 5. Conclusions

This study highlights the significant role of *Tomicus* species in the potential spread of pathogenic fungi, particularly ophiostomatoid fungi, under current environmental conditions. While the fungal communities associated with *T. piniperda* were more strongly influenced by environmental factors than by beetle species, the presence of known pathogens such as *Ophiostoma* spp., *D. sapinea*, and *Fusarium* sp. indicates that *Tomicus* species could play a crucial role in the dispersal of harmful fungi.

The varying virulence of these fungi, combined with their association with potentially pathogenic species, suggests that *Tomicus* species may contribute to forest health decline, particularly in response to changes in environmental conditions or host susceptibility. Continued monitoring and further research into the interactions between fungi and beetles are essential for a deeper understanding of the risks these interactions pose to forest ecosystems, especially as climate change and shifting host dynamics may exacerbate the spread of forest pathogens.

## Figures and Tables

**Table 1:** Insect materials collected for fungal community study.

Country		No. of Individuals	Forest Stands	City, Region	Geographical Position
Lithuania	*T. piniperda*	48	*P. sylvestris*	Kaunas (1)	54°50′13.6″ N, 24°4′ 22.1″ E
Ukraine	*T. piniperda*	96	*P. sylvestris*	Manevychy (2)	51°18′36.5″ N 25°33′17.7″ E
	*T. piniperda*		*P. sylvestris*	Ivankiv (3)	50°55′17.0″ N 29°47′42.0″ E
Montenegro	*Tomicus cf. destruens*	36	*P. halepensis*	Bar (4)	42°06′25.2″ N 19°05′17.9″ E

**Table 2:** Relative abundance of the most common fungal OTUs detected in association with pine bark beetles in Lithuania (LT), Montenegro (MN), and Ukraine (UA).

OTU	Phylum	Lifestyle	Relative Abundance %			
			LT	MN	UA	All
*Alternaria alternata* (Fr.) Keissl.	Ascomycota	Plant pathogen	11.3	-	0.8	4.03
*Ambrosiella hartigii* L.R. Batra	Ascomycota	Saprotrophic blue-stain	1	-	-	0.33
*Aureobasidium pullulans* (de Bary & Löwenthal) G.Arnaud	Ascomycota	Saprotroph	0.1	3.6	0.5	1.4
*Beauveria pseudobassiana* S.A. Rehner and Humber	Ascomycota	Entomopathogen	1.1	-	0.9	0.7
*Botrytis cinerea* Pers.	Ascomycota	Plant pathogen	0.2	0.8	0.2	0.4
*Ceratocystis* sp.	Ascomycota	Plant pathogen	0.1	-	0.05	0.05
*Cladosporium* sp.	Ascomycota	Saprotroph	29.2	12.4	22.7	21.4
*Curvibasidium cygneicollum* J.P. Samp.	Basidiomycota	Saprotrophic wood-rotter	0.3	-	0.2	0.17
*Cyberlindnera mississippiensis* (Kurtzman, M.J. Smiley, C.J. Johnson, Wick) Minter	Ascomycota	Saprotroph	-	12.5	-	4.2
*Dothiora prunorum* (C. Dennis & Buhagiar) Crous	Ascomycota	Plant pathogen	-	3.6	-	1.2
*Dothiorella sarmentorum* (Fr.) A.J.L. Phillips, A. Alves, and J. Luque	Ascomycota	Plant pathogen	0.01	-	0.5	0.17
*Dothistroma* sp.	Ascomycota	Plant pathogen	-	4.9	-	1.63
*Filobasidium magnum* (Lodder & Kreger-van Rij) Xin Zhan Liu, F.Y. Bai, M. Groenew and Boekhout	Basidiomycota	Saprotroph	-	-	0.7	0.23
*Filobasidium wieringae* (Á. Fonseca, Scorzetti & Fell) Xin Zhan Liu, F.Y. Bai, M. Groenew and Boekhout	Basidiomycota	Saprotroph	0.05	0.03	-	0.03
*Fusarium oxysporum* Schltdl.	Ascomycota	Plant pathogen	0.3	18.8	0.01	6.37
*Geosmithia* sp.	Ascomycota	Plant pathogen	-	-	0.1	0.03
*Kalmusia* sp.	Ascomycota	Plant pathogen	-	4.9	-	1.63
*Moristroma germanicum* C. Kraus, Damm, S. Bien, Vögele and M. Fisch	Ascomycota	Unassigned	0.3	-	0.5	0.27
*Ogataea pini* (Holst) Y. Yamada, M. Matsuda, K. Maeda and Mikata	Ascomycota	Saprotroph	6.8	-	-	2.27
*Ophiostoma* sp.	Ascomycota	Plant pathogen	21.8	14.6	25.4	20.6
*Penicillium spathulatum* Frisvad and Samson	Ascomycota	Saprotroph	2.9	0.5	1.8	1.73
*Peterozyma* sp.	Ascomycota	Saprotroph	18.2	8.2	10.8	12.4
*Phacidiales* sp.	Ascomycota	Plant pathogen	0.9	-	0.3	0.4
*Ramularia vizellae* Crous	Ascomycota	Plant pathogen	0.1	-	-	0.03
*Rhodotorula glutinis* (Fresen.) F.C. Harrison	Basidiomycota	Saprotroph	0.2	-	-	0.06
*Sarocladium strictum* (W. Gams) Summerb.	Ascomycota	Saprotroph	0.1	-	0.1	0.06
*Diplodia sapinea* (Fr.) Dyko and B. Sutton	Ascomycota	Plant pathogen	2.8	14.5	34.2	17.17
*Sporobolomyces roseus* Kluyver and C.B. Niel	Basidiomycota	Mycoparasite	0.05	0.06	0.04	0.05
*Stemphylium vesicarium* (Wallr.) E.G. Simmons,	Ascomycota	Plant pathogen	0.1	-	0.1	0.06
*Vishniacozyma carnescens* (Verona & Luchetti) Xin Zhan Liu, F.Y. Bai, M. Groenew and Boekhout	Basidiomycota	Saprotroph	1.1	-	-	0.37

**Table 3:** Relative abundance of the ophiostomatoid species (OTUs) associated with pine bark beetles in Lithuania (LT), Montenegro (MN), and Ukraine (UA).

OTU	Family	Relative Abundance %		
		LT	MN	UA
*Ambrosiella hartigii*	Ceratocystidaceae	1	-	-
*Ceratocystis* sp.	Ceratocystidaceae	0.1	-	0.05
*Geosmithia* sp.	Sordariomycetes	-	-	0.1
*Ophiostoma* sp. 1–5	Ophiostomataceae	21.8	14.6	25.4
*Leptographium* sp. 1, 2	Ophiostomataceae	≤0.01	≤0.01	≤0.01
*Leptographium* sp. 3	Ceratocystidaceae	-	≤0.01	≤0.01
*Graphium* sp.	Microascaceae	≤0.01	-	-

## Data Availability

The datasets generated during and/or analyzed during this study are available from the corresponding author upon reasonable request.
